# Microbiome diversity across physicochemical gradient in low-medium enthalpy springs at the Sierra Madre Oriental eastern flank, northeastern Mexico

**DOI:** 10.3389/fmicb.2025.1663000

**Published:** 2025-09-30

**Authors:** María Cruz Juárez-Aragón, Jerjes R. Pantoja-Irys, Edilia de la Rosa-Manzano, Lorena Garrido-Olvera, Hugo Mujica-Sánchez, Carlos Rafael Trejo-De León, Alejandra Vázquez-Lobo

**Affiliations:** ^1^Instituto de Ecología Aplicada, Universidad Autónoma de Tamaulipas, Ciudad Victoria, Mexico; ^2^Corporación Ambiental de México, Monterrey, Mexico; ^3^Facultad de Ingeniería y Ciencias, Universidad Autónoma de Tamaulipas, Ciudad Victoria, Mexico; ^4^Centro de Investigación en Biodiversidad y Conservación, Universidad Autónoma del Estado de Morelos, Cuernavaca, Mexico

**Keywords:** bacterial diversity, environmental factors, hot springs, Mexico, microbiome

## Abstract

**Introduction:**

Bacterial communities are fundamental to the functionality of thermal springs where they engage in essential processes such as the oxidation of sulfur, reduction of nitrates, carbon fixation, production of unique metabolites, and stabilization of microbial trophic networks. Northeastern Mexico presents a diverse array of thermal springs located within tropical karst systems situated among folded mountains and ancient inactive karstic regions. The geological complexity of these environments indicates a substantial potential for microbiome diversity; however, the composition and functional dynamics of microbial communities in these springs have not been thoroughly investigated.

**Methods:**

This study involved the collection of water samples from six hot springs, to characterize the planktonic microbiome using advanced metagenomic sequencing techniques. Additionally, we examined the relationship between microbial composition and physicochemical parameters.

**Results:**

Our analysis identified a total of 425 microbial species, which included 409 bacterial species, 13 eukaryotic organisms, and 3 archaeal taxa. The Ojo Caliente and Mainero Azufroso springs displayed the highest microbial diversity, whereas the Balneario El Bañito and Taninul springs exhibited the lowest. The Phyum Pseudomonadota was the predominant across the majority of springs, while Campylobacterota and Chlorobiota were specifically identified in the less diverse Balneario El Bañito and Taninul springs, respectively. A total of 30 indicator species were identified, predominantly in El Bañito and Potrero Prieto springs, emphasizing the distinctiveness of their microbial environments. Moreover, we found that electrical conductivity and bicarbonate concentration had a significant impact on the structure of this microbial communities.

**Discussion:**

This study highlights the ecological importance of these unique ecosystems in northeastern Mexico, with the Mainero Azufroso and Ojo Caliente springs identified as reservoirs of high microbial diversity.

## 1 Introduction

Geothermal systems represent extreme environmental conditions conducive to the establishment of microbial communities, encompassing both prokaryotic and eukaryotic organisms. These microorganisms assume critical ecological roles, such as regulating biogeochemical cycles (Sorokin et al., 2014; Martínez-Espinosa, 2020) and fostering interspecific metabolic interactions (Weiland-Bräuer, 2021). In these unique environments, microorganisms have developed a range of adaptive strategies to withstand harsh conditions, which may include acidic or alkaline pH, high salinity, elevated pressure, and extreme temperature variations (Merino et al., 2019; Ortega-Villar et al., 2024). Such adaptations have led to the specialization and formation of distinct taxonomic groups such as mesophiles, acidophiles, alkaliphiles, and thermophiles (Von Hegner, 2020; Sriaporn et al., 2023).

Key physicochemical parameters such as temperature, pH, nutrient availability, oxygen concentration, and the presence of heavy metals significantly influence microbial distribution in hot springs (Cho et al., 2016). The interplay of these factors shapes microbial community composition, constrains species diversity and affects metabolic and biochemical functions. For instance, deviations in pH from optimal levels can drastically impair mesophilic growth (Madigan et al., 2021), whereas thermophiles exhibit resilience across acidic or alkaline environments contingent on environmental stability (Martínez, 2024). Mesophilic microorganisms generally thrive under moderate conditions, typically around 37 °C, while thermophiles are adapted to elevated temperatures yet may be sensitive to extreme pH ranges, notably between 5 and 9 (Kruglikov and Xia, 2024). Acidophilic bacteria such as *Acidithiobacillus ferrooxidans* and *Leptospirillum* spp. predominately inhabit acidic environments due to their proficiency in low pH conditions and ability to facilitate mineral oxidation processes (Aliyu et al., 2024). Conversely, alkaline springs favor species such as *Bacillus alcalophilus* and halophilic archaea from the genus *Natronobacterium*, which exhibit strong adaptations to high-pH conditions.

Electrical conductivity, a metric reflecting interactions among dissolved minerals in water, emerges as a crucial determinant of microbial community stability and diversity. This parameter holds particular significance in thermal environments where the concentration of compounds such as sulfates and carbonates directly influences microbial community structures (Dong et al., 2022). Additionally, oxygen availability serves as a vital factor in microbial development, distinguishing between aerobic microorganisms that require elevated oxygen levels for metabolism, and anaerobic organisms that employ alternative electron acceptors like sulfates. Microaerophilic microbes, meanwhile, can thrive under low-oxygen conditions, demonstrating adaptability to environments with limited oxygen concentrations (Valcheva et al., 2020). Notably, further research is required to elucidate how the interaction between these gradients, local geochemical characteristics, isotopic compositions, and oxygen levels shapes microbiome diversity within geothermal systems, offering significant implications for biotechnological and ecological applications.

**Figure 1:**
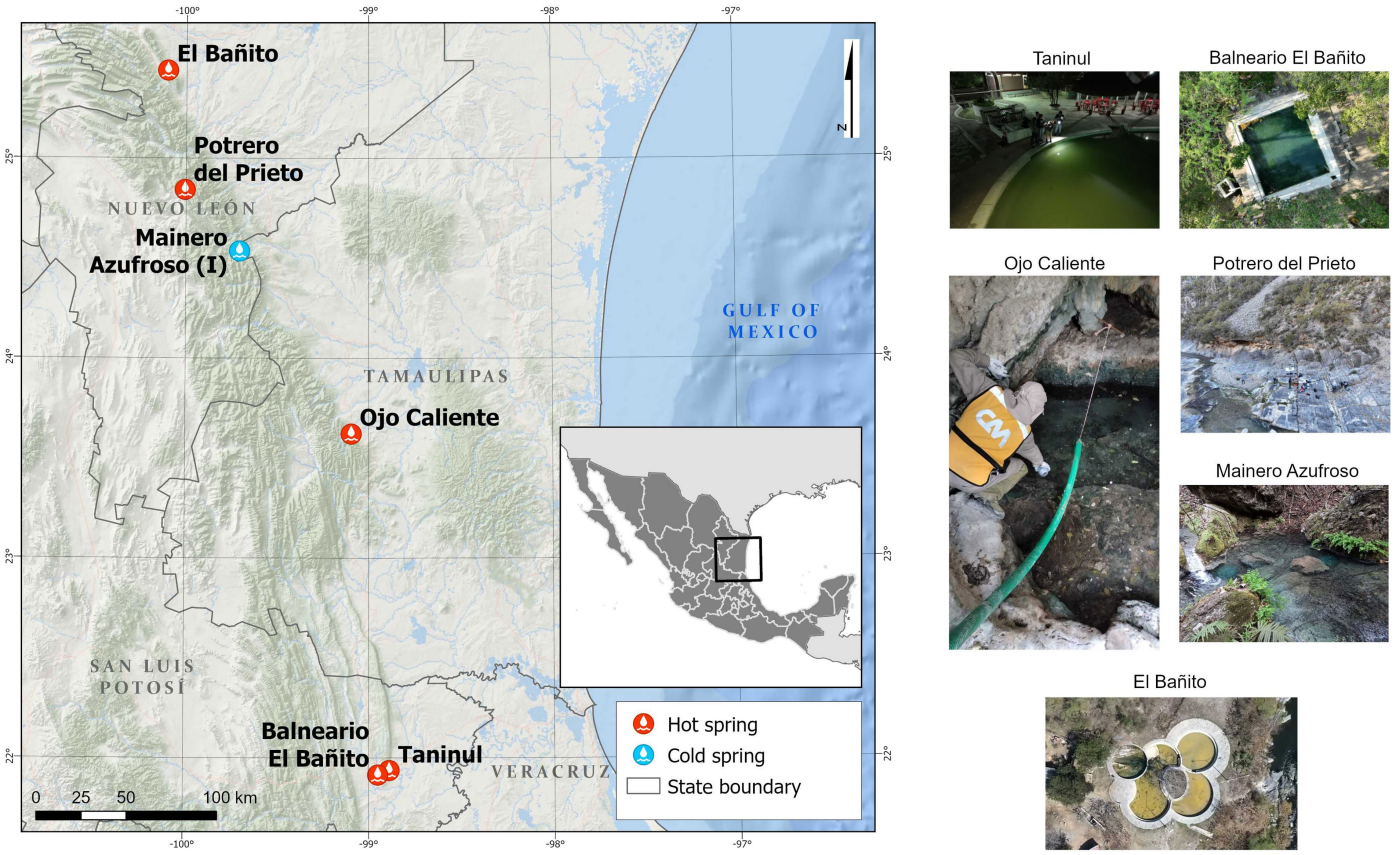
Location of hot springs in Northeastern Mexico. Potrero del Prieto, Balneario El Bañito, Taninul, El Bañito, Mainero Azufroso, and Ojo Caliente.

The hot springs in northeastern Mexico emerge from the northeastern front of the Sierra Madre Oriental (Figure 1), a NW-SE-oriented mountain belt characterized by elongated, narrow ridges serving as recharge zones for regional aquifers. These aquifers develop within tropical karst systems shaped by folded and faulted mountains in the south, and inactive karst formations in the north (Espinasa-Pereña and Nieto-Torres, 2015; Pantoja-Irys et al., 2022). Within this region, various hot springs exist, emitting hydrogen sulfide vapors that give rise to unique aquatic ecosystems marked by sulfur, gypsum, calcite or halite precipitation nearby. However, knowledge regarding the diversity patterns and taxonomic composition of the microbiomes in these geothermal springs remains limited (Castelán-Sánchez et al., 2020; Prieto-Barajas et al., 2017).

In this study, 16S rRNA gene amplicon sequencing was employed to: (1) analyze the diversity and taxonomic composition of microbial communities in thermal waters from six springs in northeastern Mexico; (2) evaluate the effects of geochemical variables on microbial community composition; and (3) identify microbial indicator species based on their ecological significance. Addressing these questions is paramount for advancing our understanding of microbial adaptability within geothermal ecosystems. The findings of this study aim to provide an integrated perspective on how microbiomes adjust to geothermal environments, establishing a baseline for the sustainable exploration and potential utilization of these invaluable resources.

## 2 Materials and methods

### 2.1 Description of low and medium enthalpy springs

This study focuses on six selected hot springs, located along the northeastern foothills of the Sierra Madre Oriental (SMO) in the Mexican states of Nuevo León, Tamaulipas, and San Luis Potosí, Mexico (Figure 1). The springs examined include: Potrero del Prieto (PP), El Bañito (EB), Ojo Caliente (OC), Taninul (TA), Balneario El Bañito (BEB), and Mainero Azufroso (MA). Notably, Mainero Azufroso, although exhibiting a cooler temperature similar to that of the adjacent stream, releases hydrogen sulfide vapors that contribute to the formation of unique aquatic ecosystems characterized by the presence of “green mats” and localized precipitation of sulfur, gypsum, calcite, or halite, warranting its inclusion in this study.

The PP hot spring is situated near the Prieta Linda waterfall and the town of El Potrero del Prieto de Arriba, nestled between the Iturbide anticline and the El Mezquital syncline in the Sierra El Baño, on the bed of the Cabezones River, at an elevation of 1,229 meters above sea level (masl). This spring is recognized as the highest hot spring in the SMO. The closest meteorological station -19073 Galeana- (Servicio Meteorológico Nacional (Mexico), 2021), reports an annual precipitation of 361.8 mm, and the region is classified as having a dry semi-warm climate (http://www.inegi.org.mx/temas/climatologia/). This hot spring emerges from the Lower Tamaulipas Formation of the Lower Cretaceous and currently has no designated use.

The EB hot spring is situated at the central part of the anticline of the sierra Cerro de La Silla, near the Rodriguez Gómez dam and the La Chueca creek at an elevation of 405 masl. The closest meteorological station -19069 La Boca- (SMN, 2021), reports an annual precipitation of 1001 mm, and the region is classified as having a semi-warm climate with summer rain (www.inegi.org.mx/temas/climatologia/). This hot spring emerges from the La Casita Formation of the Upper Jurassic and currently is used for recreational purposes.

The OC hot spring is located at an altitude of 364 masl, at the base of the El Platanillo mountain range in the Sierra El Filo. The nearest meteorological station, -28218 La Boca- (Servicio Meteorológico Nacional (Mexico), 2021), documents an average annual precipitation of 743 mm, with the area experiencing a temperate subhumid climate (http://www.inegi.org.mx/temas/climatologia/). This spring originates in the San Felipe Formation of the Upper Cretaceous and is situated on private property, currently utilized for livestock.

The TA hot spring is well-regarded locally for its medicinal and recreational applications, possibly dating back to pre-Hispanic times. It is part of a hotel complex and is located in the Sierra El Abra-Tanchipa, at the foot of the El Abra mountain range, emerging from the Cretaceous El Abra Formation at an elevation of 64 masl. The nearest meteorological station -3145 El Choy- (Servicio Meteorológico Nacional (Mexico), 2021) indicates an annual precipitation of 1165.4 mm, with the region characterized by a warm subhumid climate (http://www.inegi.org.mx/temas/climatologia/).

The BEB hot spring is currently used for recreational purposes within the municipality of Ciudad Valles. It is situated on a gently sloping hilltop, at 55 masl. According to the Ciudad Valles -24012- meteorological station (Servicio Meteorológico Nacional (Mexico), 2021) annual precipitation is reported at 1241.2 mm. This spring emerges from the San Felipe Formation, existing within a warm subhumid climate (http://www.inegi.org.mx/temas/climatologia/).

The MA spring rises at 715 masl, along the bed of an intermittent stream in the Sierra La Guitarra, at the base of the San Manuel Mountain range. The Villa Mainero −3735- meteorological station (Servicio Meteorológico Nacional (Mexico), 2021), records an annual precipitation of 993.8 mm, and the area is characterized by a temperate subhumid climate (http://www.inegi.org.mx/temas/climatologia/). This spring originates from the Taraises Formation of the Lower Cretaceous and currently lacks a specific use.

### 2.2 Physicochemical variables

At each spring location, a comprehensive assessment was conducted involving the measurement of 11 physicochemical variables: temperature, dissolved oxygen, electrical conductivity, salinity, oxidation–reduction potential, pH, turbidity, alkalinity, OH^−^, CO₃^2−^, and HCO₃^−^. The methodologies employed for the precise measurement of each variable are detailed in Pantoja-Irys et al. (2025).

### 2.3 Sample collection

Water samples were systematically collected directly from the spring source to minimize any influence from external water flows and to maintain the integrity of the native microbial communities. At each designated sampling site, five 1-liter replicates were collected using sterile plastic bottles. Upon filling, the bottles were promptly placed into a cooler to sustain a low temperature and inhibit microbial growth prior to the filtration process.

Filtration was conducted to effectively isolate and concentrate the microorganisms present in the water samples. This was achieved using sterile cellulose ester membranes with pore sizes of 0.45 μm, which are capable of retaining bacteria and other microbial cells. The filtration equipment consisted of a filtration funnel, a Kitazato flask connected to a vacuum pump, and sterilized membrane filters. During the assembly of the equipment, the membranes were meticulously positioned in the funnel using sterile gloves and dissecting forceps to prevent any risk of contamination.

The funnel was securely attached to the Kitazato flask, which was connected to a vacuum pump to establish negative pressure. Subsequently, the membranes were transferred to sterile 10 mL Falcon tubes, appropriately labeled and stored at 4 °C to preserve the DNA until subsequent analysis.

The filtered samples were shipped to MR DNA Laboratory (Shallowater, Texas, United States) for comprehensive microbial community profiling utilizing 16S rRNA gene amplicon sequencing. DNA was extracted using proprietary MR DNA protocols specifically optimized for environmental samples. The V4 region of the 16S rRNA gene was amplified using the universal primer pair 515F (GTGYCAGCMGCCGCGGTAA) and 806R (GGACTACNVGGGTWTCTAAT). PCR amplification was conductedperformed in a single-step reaction using the HotStarTaq Plus Master Mix Kit (Qiagen, USA) withemploying the following thermocycling conditions: an initial denaturation at 95 °C for 5 min; 30 cycles of denaturation at 95 °C for 30 s, annealing at 53 °C for 40 s, and extension at 72 °C for 1 min; followed by a final extension at 72 °C for 10 min. The PCR products were visualized on 2% agarose gels, pooled in equimolar ratios based on concentration and molecular weight, and purified using calibrated SPRI (Solid Phase Reversible Immobilization) beads. Sequencing was performed on the Illumina NovaSeq 6000 platform using paired-end chemistry (2 × 250 bp) according to the manufacturer’s protocols.

The sequence data were processed the MR DNA proprietary bioinformatics pipeline and QIIME2 v2023.2 (Bolyen et al., 2019). Paired-end reads were joined, and sequences shorter than 150 bp or containing ambiguous base calls were removed. Primer sequences were trimmed using Cutadapt, and reads were quality-filtered using a maximum expected error threshold of 1.0. Unique sequences were dereplicated and denoised using the UNOISE3 algorithm to generate amplicon sequence variants (ASVs), also referred to as zero-radius operational taxonomic units (zOTUs). Chimeras were detected and removed with UCHIME (Edgar et al., 2011). Taxonomic classification of ASVs was performed using BLASTn against a curated version of the NCBI nucleotide database (Edgar, 2010). The final outputs included absolute abundance tables and relative abundance matrices at various taxonomic ranks from phylum to species, as well as zOTU-to-sample mapping files (Edgar, 2016).

### 2.4 Data analysis

In order to assess species diversity within six hot spring communities, species richness and diversity were estimated using Hill numbers of order q = 0, 1 and 2. Hill numbers offer a comprehensive framework for quantifying diversity based on the effective number of species, with variations contingent on the parameter q. This parameter allows for an adjustment in the weighting of species abundances, effectively reflecting diverse aspects of community structure and enabling comparisons across samples with distinct dominance patterns (Jost, 2006; Moreno et al., 2011). Specifically, species richness (q = 0) quantifies the total number of species, without regard to their abundances. Shannon diversity (q = 1) provides a balanced estimate that incorporates both richness and evenness, thereby moderately weighting species according to their relative abundances. In contrast, Simpson diversity (q = 2) places greater emphasis on the most abundant species, consequently reducing the influence of rare species and effectively highlighting patterns of dominance within the community.

Inventory completeness was standardized through the use of sample coverage (Ĉn) facilitating a meaningful comparison of spring diversity, across various communities and ensuring that all were analyzed at consistent level of sampling completeness (Chao and Jost, 2012; Chao and Hsieh, 2016). Effective diversity estimates and sample coverage were derived utilizing the “iNEXT” function from the iNEXT package in R (https://www.r-project.org/). Statistical comparisons were conducted on the 95% confidence intervals of the Hill numbers, where significant differences were inferred if the 95% confidence intervals did not overlap.

Additionally, spring communities were classified based on species composition using a cluster dendrogram constructed using the Bray–Curtis dissimilarity index and Ward’s agglomeration method. Given the sensitivity of the Bray–Curtis index to species abundances, bacterial species abundances were log-transformed (x + 1) prior to analysis to mitigate this influence and to achieve a balanced representation of both common and rare species. The analysis was performed using the “hclust” function from the vegan package in R.

A Principal Component Analysis (PCA) was performed to identify the physicochemical variables associated with the variance among community groups observed in the studied springs. Prior to conducting the analysis, we examined the correlations among variables, identifying pairs with high correlation coefficients (> 0.85). Representative variables were selected and subsequently log-transformed, with the exception of pH, which is inherently expressed on a logarithmic scale. The PCA facilitated a visualization of the influence of environmental variables on the spring groups, allowing us to and to discern key environmental gradients. The analysis was executed using the “rda” function from the *vegan* package in R, resulting in a two-dimensional ordination plot. In this plot, community groups are represented as points, while physicochemical variables are denoted as vectors (arrows). The length and direction of each vector indicate the magnitude and direction of influence exerted by the respective variable. Groups situated near the terminus of the vectors exhibit a strong association with corresponding environmental gradients, whereas those positioned closer to the origin exhibit reduced influence from the measured variables.

Additionally, we estimated the indicator values of species within the community groups to identify the most robust and ecologically relevant indicator species for each group. To enhance the reliability of this analysis, we filtered the dataset used in the clustering analysis to include only 168 species, which collectively represented 95% of the total abundance. This approach allowed us to concentrate on species that significantly contribute to community structure. The indicator value for each species was calculated using the method proposed by Dufrêne and Legendre (1997) known as *IndVal*. This method quantifies both the specificity and fidelity of each species to a particular habitat, where specificity denotes the exclusivity of a species to a given group, and fidelity refers to the frequency of occurrence within that group. Indicator values were calculated independently for each taxon and expressed as percentages, yielding a robust metric for assessing species associations with various spring types. The analysis was conducted using the statistical software PAST version 4.17 (https://www.nhm.uio.no/english/research/resources/past/).

## 3 Results

### 3.1 Physicochemical environment

The TA hot spring exhibited the highest temperature and turbidity among the studied springs, while the PP spring demonstrated the highest salinity levels. Although no significant thermal anomaly was identified in the MA spring, it recorded the highest concentration of dissolved oxygen (Table 1). All springs maintained a neutral pH, and generally exhibited high alkalinity, predominantly in the form of bicarbonate, along with a negative redox potential, with the exception of the OC spring (Table 1).

**Table 1:** Characterization of the physicochemical variables of hot springs in Northeastern Mexico.

Spring	T (°C)	DO (mg/L)	EC (μS/cm)	Sal (ppt)	ORP (mV)	pH	Turb (NTU)	Alk (mg/L)	OH(−) (mg/L)	CO_3_(2−) (mg/L)	HCO_3_(−) (mg/L)
Taninul	38.00	0.05	1,671	0.58	−7.40	6.52	38.36	350.63	0	0.40	426.68
Balneario El Bañito	32.60	1.22	1,102	0.47	−154.70	7.27	7.20	259.63	0	0.20	314.73
Ojo Caliente	31.30	1.29	1,049	0.46	70.00	6.82	0.00	217.35	0	0.10	264.78
Potrero del Prieto	26.30	0.81	2,421	1.21	−247.90	6.94	0.00	160.83	0	0.10	195.83
Mainero Azufroso	19.90	3.56	362	0.19	−150.50	7.4	0.00	182.07	0	0.15	221.73
El Bañito	35.2	3.67	1,309	0.53	182.6	6.89	0.35	190	<1	<1	190

DO, dissolved oxygen; EC, electrical conductivity; Sal, salinity; ORP, oxidation–reduction potential; Turb, turbidity; Alk, alkalinity.

### 3.2 Taxonomic composition

The microbiome of the six hot springs comprised 425 species, with the majority (409) attributed to the domain Bacteria, alongside 13 to Eukarya, and 3 to Archaea. The bacterial dataset encompassed 177,136 counts across the six springs, distributed among 31 phyla, 43 classes, 73 orders, 138 families, 245 genera, and 409 species. Notably, the highest bacterial counts were recorded in the TA, BEB, and PP springs, with 32,718, 31,580, and 30,921 reads, respectively. Conversely, the MA, the OC, and the EB springs exhibited the lowest counts, with 26,301, 27,803, and 27,813 reads, respectively.

The bacterial communities within the MA and the OC springs displayed the highest taxonomic richness across all taxonomic levels, from phylum to species. In contrast, the TA and the BEB springs exhibited the lowest overall taxonomic representation. While the BEB and the PP springs contained a greater number of sequence-assigned species compared to the TA spring, both communities exhibited lower richness at the phylum level, indicating a more constrained phylogenetic breadth. It is noteworthy that despite a similar number of species in the MA and OC springs, the MA spring was characterized by a higher richness at the phylum level, suggesting a broader phylogenetic diversity (Table 2).

**Table 2:** Taxonomic composition of bacterial communities recorded in six hot springs from Northeastern Mexico.

Taxa	PP	BEB	TA	EB	MA	OC
Phyllum	14	13	16	18	25	19
Class	20	18	19	22	33	29
Order	41	38	25	41	55	50
Family	80	69	35	75	104	92
Genus	131	107	42	134	172	168
Species	215	153	47	206	285	289

PP, Potrero del Prieto; BEB, Balneario El Bañito; TA, Taninul; EB, El Bañito; MA, Mainero Azufroso; OC, Ojo Caliente.

**Figure 2:**
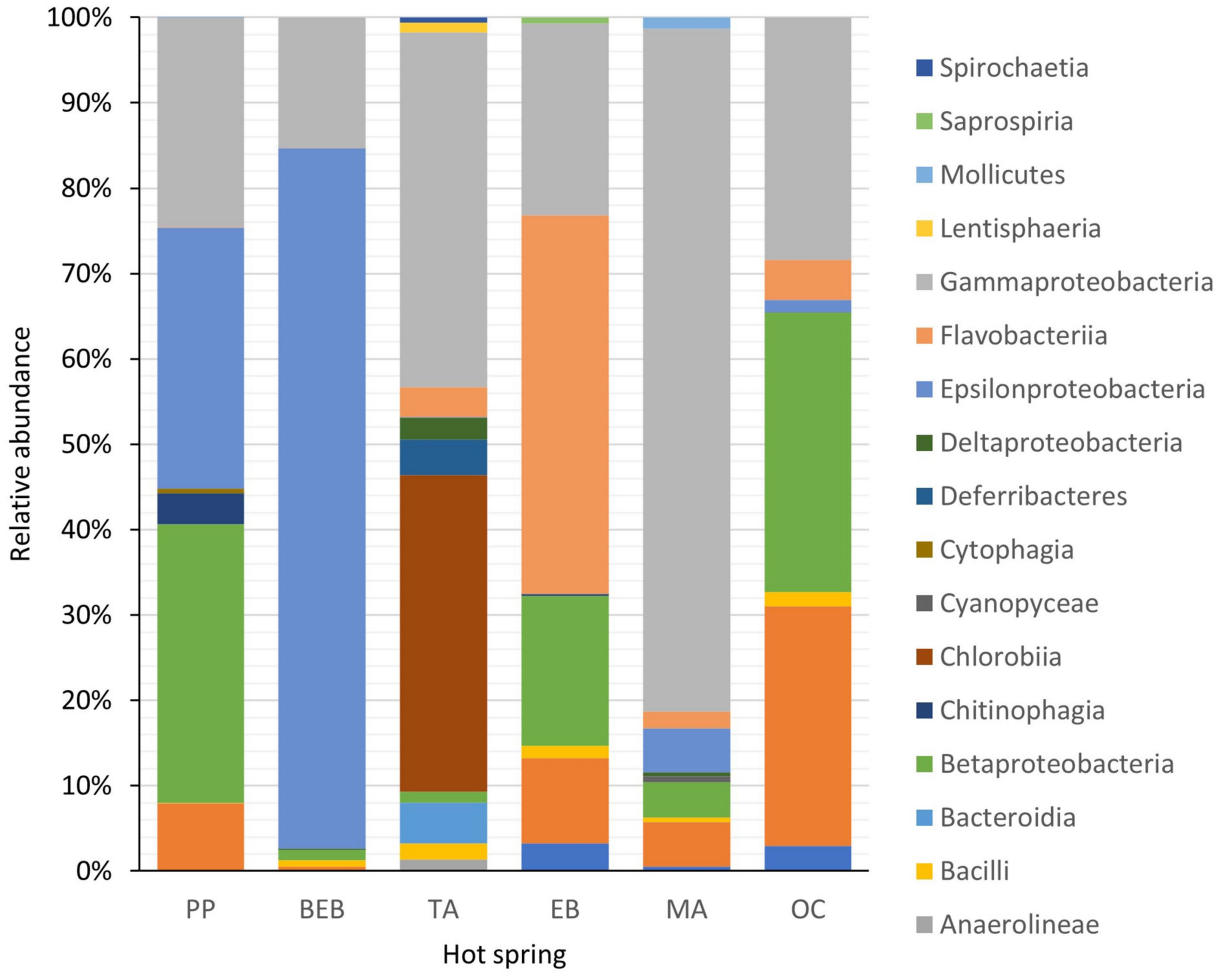
Relative abundance of 17 bacterial phyla recorded in six hot springs located in Northeastern Mexico. PP, Potrero del Prieto; BEB, Balneario El Bañito; TA, Taninul; EB, El Bañito; MA, Mainero Azufroso; OC, Ojo Caliente.

The phylum Pseudomonadota was found to be the most abundant across all springs with the exception of the BEB and the TA springs, where Campylobacterota and Chlorobiota, respectively, dominated. Additionally, Bacteroidota ranked as the second most abundant phylum within spring EB (Figure 2).

The taxonomic diversity observed in the investigated ecosystems revealed several dominant classes, notably Gammaproteobacteria, Betaproteobacteria, Epsilonproteobacteria, Chlorobiia, Flavobacteriia, and Alphaproteobacteria. Epsilonproteobacteria was particularly prevalent in the BEB spring, representing 80.2% of the total microbial abundance. Similarly, Gammaproteobacteria showed significant abundance in the MA spring, accounting for 71.5% of the total, while Flavobacteriia was the predominant group in the EB spring, contributing 40% to the overall abundance. It is noteworthy that Chlorobiia was exclusively identified in the TA spring, where it constituted 36.3% of the total microbial population.

At the order level, key representative groups included Campylobacterales in both the PP and BEB springs; Chromatiales and Chlorobiales in the TA spring; Flavobacteriales in the EB spring; Chromatiales again in the MA spring. In the OC spring, the dominant orders were and Burkholderiales, Moraxellales, and Rhodobacterales in the OC spring.

A detailed analysis of representative families and genera across the springs reveals that in the PP spring, Arcobacteraceae and Chromobacteriaceae were predominant, with the genera *Halarcobacter* and *Vogesella*; the BEB spring was characterized by Sulfurovaceae and Thiovulaceae, with *Sulfurovum* and *Sulfuricurvum* identified; the TA spring exhibited Halothiobacillaceae and Chlorobiaceae, featuring *Thiofaba* and *Chlorobaculum*; in the EB spring, Flavobacteriaceae, particularly *Flavobacterium* was prevalent; while the MA spring showcased, Halothiobacillaceae, represented by *Thiofaba*; finally, in the OC spring was marked by Comamonadaceae and Moraxellaceae, including *Limnohabitans* and *Acinetobacter*.

### 3.3 Effective diversity profile

**Figure 3:**
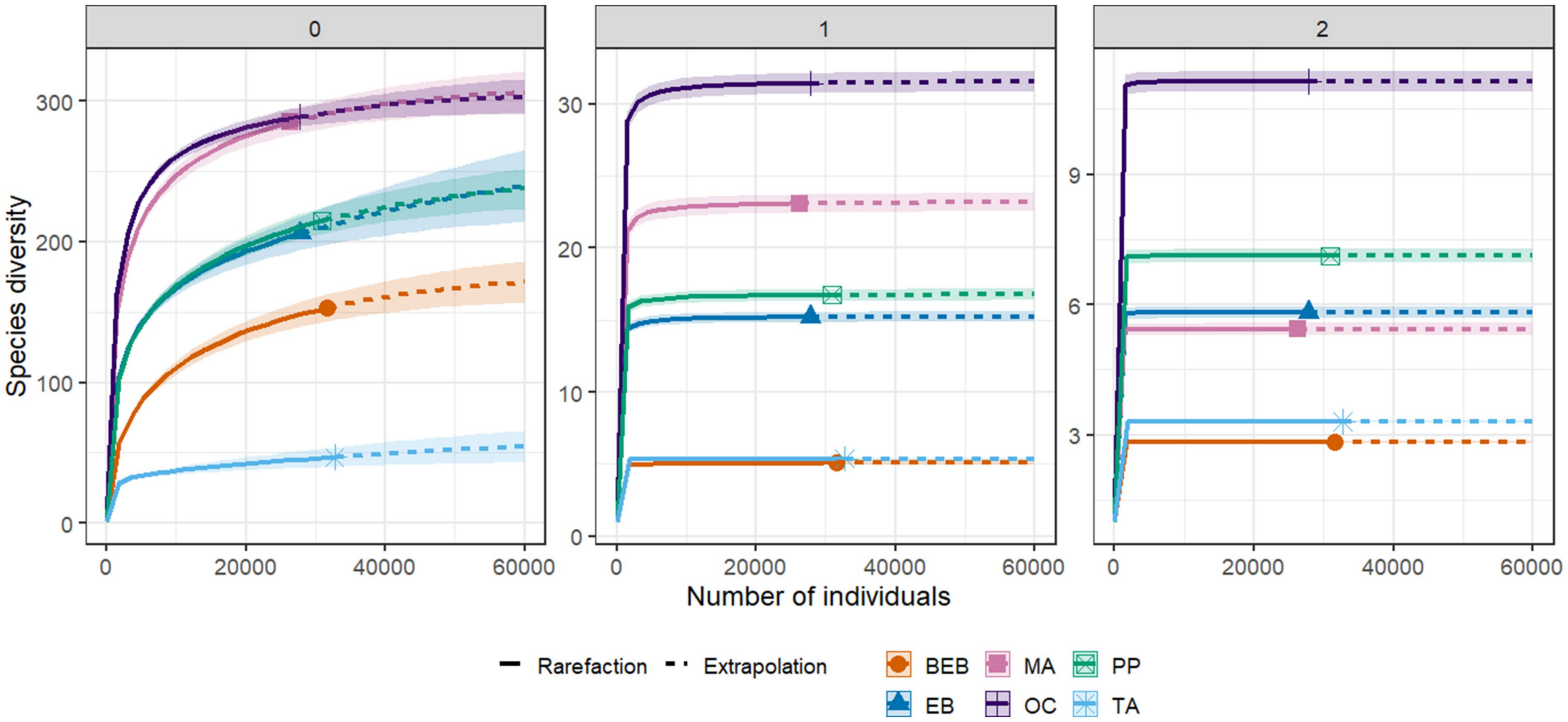
Diversity profile of bacterial communities in six hot springs from Northeastern Mexico. 0: species richness; 1: Shannon diversity; 2: inverse Simpson diversity. PP, Potrero del Prieto; BEB, Balneario El Bañito; TA, Taninul; EB, El Bañito; MA, Mainero Azufroso; OC, Ojo Caliente.

The sample coverage across all six springs surpassed 99%, with values ranging from 0.9985 to 0.9997; (Table 3). Notably, species richness (q = 0) demonstrated significant variation among springs. Springs OC and MA displayed the highest levels of bacterial richness compared to the other sites. While no statistically significant differences in species richness were detected between the PP and the EB springs, both sites exhibited greater richness compared to the BEB and the TA springs. The TA spring recorded the lowest level of richness (Table 3; Figure 3).

**Table 3:** Sample coverage (Ĉn), species richness (q0), common species (q1), and dominant species (q2) with their confidence intervals (±CI) for bacterial communities recorded in six hot springs from Northeastern Mexico.

Spring	*Ĉn*	q0	±IC	q1	±IC	q2	±IC
PP	0.9987	210.24	11.84	16.72	0.33	7.12	0.12
BEB	0.9989	143.96	9.81	5.07	0.08	2.83	0.03
TA	0.9997	32.18	1.78	5.35	0.08	3.30	0.04
EB	0.9985	206	20	15.20	0.38	5.81	0.14
MA	0.9987	280.92	12.79	23.07	0.53	5.43	0.13
OC	0.9992	276.87	7.42	31.36	0.74	11.13	0.26

PP, Potrero del Prieto; BEB, Balneario El Bañito; TA, Taninul; EB, El Bañito; MA, Mainero Azufroso; OC, Ojo Caliente.

In terms of Shannon diversity (q = 1), the OC spring exhibited the highest value among all studied springs, with a q1 = 31.36. This was followed, in descending order, by the MA, the PP, and the EB springs (Table 3; Figure 3). In contrast, the BEB and the TA springs, which demonstrated similar diversity values, recorded the lowest levels of diversity, indicating a reduced evenness in species abundance. For inverse Simpson diversity (q = 2), the OC spring again represented the apex of diversity, achieving a q2 = 11.13, while the BEB spring marked the lowest at q2 = 2.83, suggesting a pronounced dominance of a select few species. The PP, the EB, and the MA springs displayed moderately high diversity values, reflecting varied degrees of evenness in species distribution (Table 3). Notably, despite the TA spring’s relatively low species richness, its abundances were distributed more evenly than those observed in the BEB spring (Table 3).

### 3.4 Community classification

**Figure 4:**
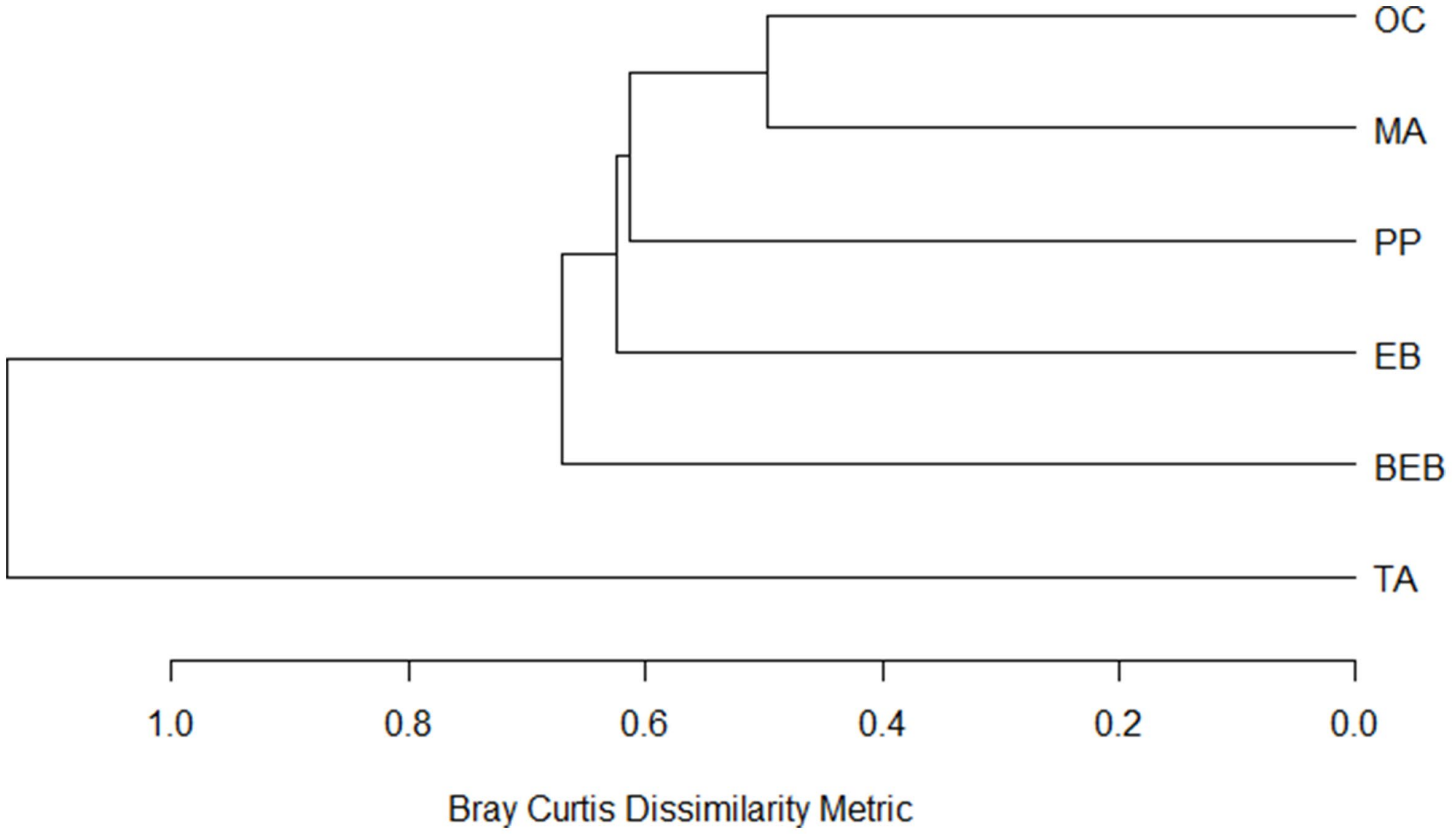
Dissimilarity dendrogram of bacterial communities from six hot springs in Northeastern Mexico. PP, Potrero del Prieto; BEB, Balneario El Bañito; TA, Taninul; EB, El Bañito; MA, Mainero Azufroso; OC, Ojo Caliente.

The classification of springs based on species composition revealed five distinct groups (Figure 4). Group I was exclusively comprised of the TA spring, while the BEB, EB, and PP springs formed separate clusters, designated as Groups II, III, and IV, respectively. The OC and MA springs were classified together in Group V, exhibiting the highest similarity in bacterial community composition (51%).

### 3.5 Community groups and their relationship to physicochemical properties

**Figure 5:**
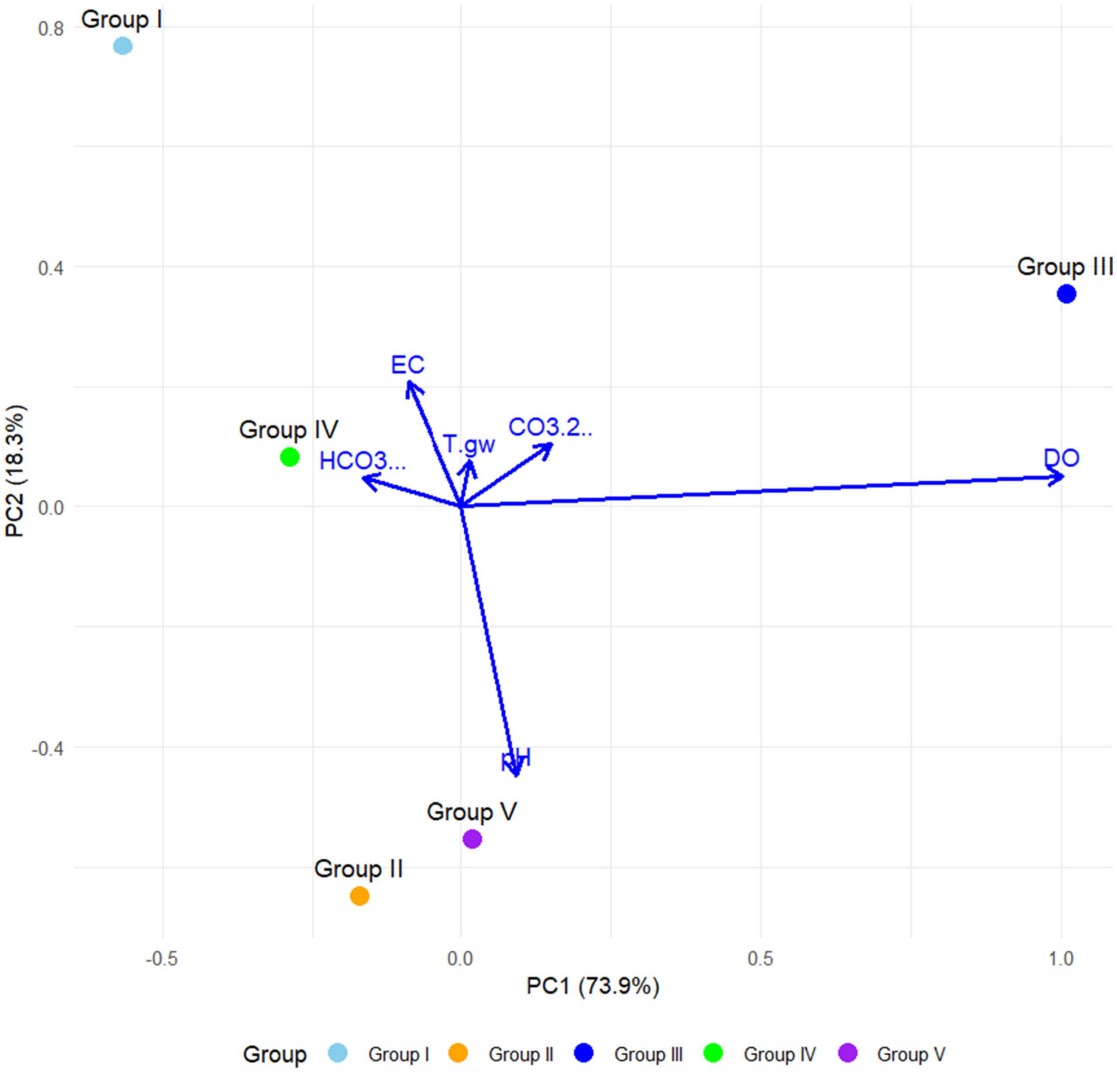
Two-dimensional principal component analysis (PCA) plot showing the physicochemical variables associated with community groups of sampled hot springs from Northeastern Mexico.

The Principal Component Analysis (PCA) revealed that the first two components accounted for 92.2% of the total variation in the dataset (Figure 5). The first principal component (PC1) accounted for 73.9% of the variability and was significantly influenced by electrical conductivity (EC) and bicarbonates (HCO₃^−^). The second principal component (PC2) accounted for 18.3% of the variation, with dissolved oxygen (DO) and pH contributing to its high values. Groundwater temperature (T°gw) had a moderate influence on PC2, although to a lesser extent compared to the other key other variables (Figure 5).

Group I demonstrated a strong association with elevated levels of HCO₃^−^ and EC along PC1. Group II was positioned near the center of the plot, indicating intermediate values across the majority of physicochemical variables. Conversely, Group III exhibited a pronounced correlation with pH and DO, suggesting conditions characterized by higher oxygen availability and a neutral to basic pH. Group IV displayed some relationship with T°gw, although no other physicochemical variable appeared to exert a dominant influence. Lastly, Group V was located near the origin, indicating more balanced or less differentiated physicochemical characteristics across the measured variables (Figure 5).

### 3.6 Indicator species

A total of 98 species were identified as having indicator potential (IndVal ≥ 50%), with 30 species demonstrating statistical significance as indicators for the various groups (Table 4). Notably, species classified as indicators in each spring exhibited over 97% homology with existing sequences.

**Table 4:** Indicator value (IndVal %) and statistical significance (p(raw)) of 30 indicator species in four bacterial community groups from Northeastern Mexico.

Species	Group I TA		Group II BEB		Group III EB		Group IV PP		Group V OC + MA	
	IndVal %	p(raw)	IndVal %	p(raw)	IndVal %	p(raw)	IndVal %	p(raw)	IndVal %	p(raw)
*Arthrobacter globiformis*	0	1	16.71	0.5469	42.45	**0.0179**	8.778	0.7157	32.06	0.2039
*Acinetobacter lwoffii*	0	1	19	0.563	44.13	**0.0179**	13.08	0.7363	23.79	0.3385
*Asticcacaulis solisilvae*	0	1	15.22	0.5469	49.64	**0.0179**	0	1	35.15	0.2039
*Brucella abortus*	0	1	8.039	0.7334	20.78	0.4662	46.89	**0.035**	24.29	0.3548
*Brevundimonas aurantiaca*	0	1	11.43	0.5538	7.209	0.7145	45.04	**0.0178**	36.32	0.2027
*Cupriavidus cauae*	0	1	23.28	0.2478	63.75	**0.0495**	0	1	12.96	0.537
*Delftia acidovorans*	0	1	0	1	47.51	**0.0179**	15.4	0.5541	37.08	0.2039
*Dyella ginsengisoli*	0	1	67.93	**0.0358**	7.674	0.4994	0	1	24.39	0.3386
*Delftia litopenaei*	0	1	0	1	62.8	**0.0348**	14.98	0.4313	22.22	0.3357
*Ensifer adhaerens*	0	1	15.52	0.7046	17.28	0.5685	41.64	**0.0178**	25.55	0.3325
*Exiguobacterium aurantiacum*	0	1	0	1	62.7	**0.0179**	8.172	0.5919	29.13	0.2685
*Enterococcus dispar*	45.81	**0.0492**	20.01	0.4316	14.95	0.6525	0	1	19.22	0.4627
*Erythrobacter donghaensis*	6.045	0.7519	0	1	15.62	0.4838	61.27	**0.0255**	17.06	0.4215
*Flavobacterium celericrescens*	0	1	0	1	66.57	**0.0327**	15.86	0.4387	17.57	0.4728
*Hydrogenophaga atypica*	0	1	4.596	0.7483	45.56	**0.0327**	21.6	0.3831	28.24	0.336
*Halarcobacter bivalviorum*	0	1	27.01	0.4216	0	1	52.1	**0.0351**	20.88	0.3363
*Hydrogenophaga soli*	0	1	0	1	45.27	**0.0496**	34.53	0.2603	20.2	0.4032
*Imtechium assamiensis*	0	1	7.89	0.5801	72.75	**0.0179**	0	1	19.36	0.4053
*Limnobacter alexandrii*	0	1	0	1	23.03	0.4169	54.39	**0.035**	22.58	0.3343
*Lysobacter daecheongensis*	0	1	0	1	16.57	0.5319	61.53	**0.0178**	21.9	0.2673
*Lactococcus garvieae*	63.97	**0.0176**	7.519	0.5838	0	1	0	1	28.51	0.2656
*Luteimonas lutimaris*	0	1	0	1	14.52	0.4334	55.75	**0.035**	29.73	0.3343
*Luteolibacter yonseiensis*	0	1	0	1	79.06	**0.0179**	0	1	20.94	0.4053
*Paracidovorax avenae*	0	1	0	1	74.2	**0.0179**	6.802	0.5919	18.99	0.4053
*Pseudomonas guguanensis*	0	1	0	1	54.94	**0.0348**	22.28	0.4313	22.78	0.3357
*Pseudomonas mosselii*	0	1	39.18	**0.018**	18.65	0.5484	9.852	0.7138	32.32	0.1997
*Planomicrobium okeanokoites*	0	1	17.23	0.4334	44.47	**0.0335**	9.725	0.7513	28.58	0.3372
*Pseudomonas parafulva*	0	1	13.96	0.7665	35.3	**0.0496**	26	0.2554	24.74	0.4032
*Serratia marcescens*	0	1	59.65	**0.018**	10.92	0.5828	0	1	29.43	0.2697
*Vogesella urethralis*	5.175	0.9147	13.06	0.7334	15.7	0.5685	41.71	**0.0178**	24.36	0.3325

PP, Potrero del Prieto spring; BEB, Balneario El Bañito spring; TA, Taninul spring; EB, El Bañito spring; MA, Mainero Azufroso spring; OC, Ojo Caliente spring. Bold values indicate species with significant differences (*p* < 0.05) in each spring according to the indicative value analysis (IndVal).

In Group I, 13 species showed indicator potential; however, only two species, *Enterococcus dispar* (IndVal = 45.81%, *p* = 0.0492) and *Lactococcus garvieae* (IndVal = 63.97%, *p* = 0.0176) reached statistical significance.

Group II comprised four species with indicator potential, three of which were statistically significant (Table 4). Among these, *Dyella ginsengisoli* (IndVal = 67.93%, *p* = 0.0358) and *Serratia marcescens* (IndVal = 59.65%, *p* = 0.018) were particularly noteworthy.

Group III exhibited the highest number of significant indicator species, with 21 species showing indicator potential, of which 16 were statistically significant (Table 4). Strongly associated species in this group included *Luteolibacter yonseiensis* (IndVal = 79.06%, *p* = 0.0179), *Paracidovorax avenae* (IndVal = 74.2%, *p* = 0.0179), and *Imtechium assamiensis* (IndVal = 72.74%, *p* = 0.0179).

Group IV revealed a considerable number of potential indicators, totaling 20 species, among which nine were statistically significant. Highlighted species included *Lysobacter daecheongensis* (IndVal = 61.53%, *p* = 0.0178), *Erythrobacter donghaensis* (IndVal = 61.27%, *p* = 0.0255), and *Luteimonas lutimaris* (IndVal = 55.75%, *p* = 0.035).

Lastly, Group V comprised 40 species with indicator potential; however, no species were identified as statistically significant indicators.

## 4 Discussion

This study presents the first comprehensive analysis of microbial diversity and community composition in six hot springs located along the eastern flank of the SMO in northeastern Mexico, a region distinguished by its geological complexity and unique environmental conditions. Utilizing 16S rRNA gene sequencing, we identified 425 microbial species, predominantly belonging to the domain *Bacteria*. These species are classified across 31 phyla, 43 classes, 73 orders, 138 families, 245 genera, and 409 species.

The investigation of these ecosystems is particularly valuable due to their distinctive physicochemical conditions and relatively undisturbed environments, contrasting sharply with other hydrothermal systems influenced by anthropogenic activity (López-Sandoval et al., 2016). In nearby locations, such as the Cuatro Ciénegas Basin in Coahuila, Mexico, 325 metagenome-assembled genomes have been characterized: 277 from Bacteria and 48 from Archaea, representing 40 phyla (32 bacterial and 8 archaeal). This microbial diversity is attributed to extreme environmental conditions, including high salinity, variable pH levels (ranging from 5 to 9.8), and a significant nutrient imbalance. These factors create numerous ecological niches that likely drive the endemism of microbial lineages and foster remarkable adaptations to oligotrophic conditions (Rodríguez-Cruz et al., 2024; Medina-Chávez et al., 2025). Consequently, the extensive microbial diversity documented in this study contributes to a deeper understanding of biogeographic patterns in geothermal environments and underscores their promising biotechnological potential, particularly in applications related to bioremediation and biodegradation.

While the observed microbial composition here shares certain similarities with other thermal systems studied both in Mexico and internationally, it also shows remarkable differences. For instance, in contrast to the geothermal springs in Araró, Michoacán, Mexico (Prieto-Barajas et al., 2017), where *Firmicutes* are predominant, or those in Chignahuapan, México (Castelán-Sánchez et al., 2020) which are dominated by Actinobacteria and Proteobacteria, our findings indicate that Pseudomonadota was the predominant phylum in most of the springs examined. Exceptions include the BEB and TA springs, where Campylobacterota and Chlorobionta, were more prevalent, respectively. This suggests that specific adaptations to local physicochemical conditions, such as elevated temperature and turbidity, distinctly differentiate these springs from others. The presence and relative abundance of bacterial phyla including Pseudomonadota, Campylobacterota, Chlorobiota, and Bacteroidota in geothermal springs appear closely associated with the extreme environmental conditions present.

Pseudomonadota is frequently documented in geothermal systems and exhibits remarkable adaptability across various environmental gradients. For instance, studies in Eritrea, northeastern Africa, identified genera such as *Pseudomonas* and *Marinobacter* associated with high sodium and calcium concentrations (Ghilamicael et al., 2017). Likewise, research in Julong, China, *Pseudomonas* accounted for 72% of the bacterial diversity in thermal water samples (Wang and Pecoraro, 2021). The prevalence of this genus in the MA spring may be attributed to the favorable salinity and pH conditions unique to this environment (Table 1).

Campylobacterota, encompasses chemolithotrophic bacteria that thrive in sulfur-rich environments characterized by moderate temperatures (Sun et al., 2023). Their notable abundance of this group in the BEB spring is consistent with findings from the eolian archipelago in Italy, where Arcobacteraceae predominated in spring sediments within the temperature range of 40 °C to 53 °C (Barosa et al., 2023).

Chlorobiota exclusively identified in the TA spring, is a significant discovery potentially linked to its distinct geochemical profile. These bacteria are anaerobic phototrophs typically inhabiting sulfur-rich, oxygen-poor geothermal springs, playing critical roles in carbon and sulfur cycling (Madigan et al., 2017). Chlorobiota includes anoxygenic phototrophs of the order Chlorobiales, which have been demonstrated to significantly contribute to carbon fixation in oligotrophic geothermal systems such as those in Odisha, India (Badhai et al., 2015).

Bacteroidota was also detected across several springs and is generally associated with organic matter-rich environments. In the EB spring, *Flavobacteriales* were predominant, comprising bacteria frequently reported in extreme environments characterized by high temperatures, variable pH, and mineralization. Notably, the genus *Flavobacterium* is recognized for its capability to degrade biopolymers such as cellulose, chitin, and proteins, thereby facilitating nutrient availability for other microbial taxa (Seo et al., 2024).

The 16S sequencing technique facilitated an in-depth profiling of microbial communities, effectively addressing the limitations inherent to traditional culture-based methods (Briggs et al., 2014). However, when compared to more advanced techniques such as shotgun metagenomics or PhyloChip analysis (e.g., Hamady et al., 2010), sequencing may underestimate the total microbial diversity by overlooking low-abundance or unculturable species. Remarkable, 55 bacterial taxa in the MA, 24 in the OC, and 21 in PP spring could not be classified even at the genus level, exhibiting less than 95% sequence homology with existing databases. This finding emphasizes the exceptional and largely undescribed microbial diversity in the northeastern Mexico, along with its potential for novel metabolic function.

The microbial diversity observed in the MA and OC springs was notably higher compared to the TA and the BEB springs which exhibited lower levels of diversity. This discrepancy may be attributed to temperature, which is a crucial determinant of microbial diversity. The TA hot spring, characterized by its high temperature (30 °C), turbidity, alkalinity, elevated HCO3 concentration, and low pH, demonstrated a high abundance of microbial life yet a markedly low diversity. An inverse relationship between temperature and microbial diversity has been documented in similar circumneutral to alkaline hot springs worldwide (Sharp et al., 2014; Chan et al., 2017; Narsing Rao et al., 2021). Our study highlights a clear ecological partitioning of microbial communities across the springs, influenced by localized environmental gradients and distinct geochemical profiles. Despite being situated within a common geological framework (geothermal reservoirs within evaporitic-carbonate rocks), each site maintained a taxonomically and functionally unique assemblage, highlighting the sensitivity of the microbiomes to subtle physicochemical variations.

While it is generally observed that higher temperatures correlate with reduced microbial diversity, exceptions do exist. The SK spring in Malaysia, for instance, displayed significant diversity despite elevated temperatures, likely attributed to site-specific physical conditions (Chan et al., 2017). The dominance of Gammaproteobacteria in the MA spring (71.5%) and Epsilonproteobacteria in the BEB spring (80.2%) further illustrates the significant impact of local physicochemical conditions on microbial structure. These findings align with previous studies that link *Proteobacteria* abundance to nutrient-rich, sulfurous and extreme environments (Hou et al., 2013; Castelán-Sánchez et al., 2020; Susanti et al., 2025).

Notably, the OC spring exhibited not only high species richness but also substantial evenness in species distribution, as evidenced by consistently high q1 (Shannon diversity) and q2 (inverse Simpson diversity) values. This pattern suggests the microbial community is characterized by a balanced distribution of species, with no single taxon or small group predominating. The relative evenness in species abundances indicates a diverse community structure. The OC, with its intermediate temperature (31 °C), may offer greater ecological niche availability or microhabitat diversity, thereby fostering bacterial coexistence and contributing to a more diverse and evenly structured community.

The biotechnological potential of microbial communities is significant, particularly with genera such as *Flavobacterium* and *Acinetobacter* which are recognized for their abilities to degrade organic compounds and exhibit resistance to heavy metals. These characteristics indicate promising applications in bioremediation, especially in environments contaminated with arsenic or high salinity levels (Abed and Koster, 2005).

The Principal Component Analysis (PCA) revealed substantial correlations between physicochemical parameters and the structure of microbial communities. Notably, electrical conductivity (EC) and bicarbonate (HCO₃^−^) levels have a marked influence on communities classified within Group I (the TA spring), while dissolved oxygen (DO) and pH were more intricately associated with Group III (the EB spring). Extreme pH conditions may favor the development of more specialized, less diverse communities (Guo et al., 2021), while high concentrations of salts and ions, including sodium, magnesium, chloride, and sulfate, enhance the prevalence of halotolerant or halophilic species.

Furthermore, elements such as arsenic, iron, and manganese have been observed to impose limitations on microbial diversity due to their toxic effects; however, certain bacteria have evolved resistance mechanisms to mitigate these challenges (Prieto-Barajas et al., 2017). Factors such as total organic carbon, nitrogen, and the C: N ratio are also pivotal in determining microbial growth efficiency and, consequently, diversity. This phenomenon has been documented in both the Araró region (Prieto-Barajas et al., 2017) and Malaysian springs (Chan et al., 2017), where phosphorus availability was a critical determinant of community structure. These findings indicate that local geochemical conditions play a regulatory role in shaping microbial diversity, as previously documented in other hydrothermal systems (Purcell et al., 2007). Variability in bacterial composition across different springs may be attributed to variations in salinity, temperature, and pH, which collectively influence the abundance of specific phyla such as Chlorobiota and Campylobacterota.

Indicator species analysis has identified taxa that exhibit ecological functions congruent with the physicochemical gradients present, including pH, electrical conductivity, bicarbonates, and dissolved oxygen. Notably, *Sulfurovum lithotrophicum*, a mesophilic, microaerophilic chemolithotroph, oxidizes reduced sulfur compounds such as thiosulfate and hydrogen sulfide through the Sox pathway and sulfuroquinone reductase (SQR). This species is typically found in springs characterized by elevated sulfate and hydrogen sulfide concentrations, thriving under such conditions, and playing a key role in sulfur cycling. Its detection in high-conductivity, sulfate-rich environments like BA spring corroborates its function as a bioindicator of sulfur oxidation (Wang et al., 2023), emphasizing the functional and dynamic diversity of these ecosystems.

Similarly, *Hydrogenophaga* species oxidize molecular hydrogen via hydrogenases, using oxygen or nitrate as terminal electron acceptors. This metabolic adaptation facilitates survival in microaerophilic or low redox environments (Howells et al., 2022). In the BA spring, the prevailing low redox potential and reduced dissolved oxygen create conductive conditions for this metabolic process. Moreover, several species exhibit tolerance to heavy metals such as chromium and arsenic, attributes that are increasingly leveraged in bioremediation and electrochemical reduction of Cr(VI) (Beretta et al., 2024). These functional traits highlight the significance of indicator species analysis in elucidating how hydrogen and sulfur cycling influence microbial communities and propose various environmental, energy, and biotechnological applications (Thai et al., 2023; Tang et al., 2024; Qattan, 2025).

In the MA spring it has been reported that some species of the genus *Thioalkalimicrobium* possess metabolic adaptations specialized for sulfur oxidation under alkaline conditions. These metabolic characteristics elucidate their prevalence in springs exhibiting high pH and carbonate concentrations therefore, reinforcing their role in the biogeochemical cycles. This observation aligns with literature that highlights their metabolic specialization in alkaline-sulfur environments (Sun et al., 2020; Whaley-Martin et al., 2023), potentially attributable to the mineralogical composition of the caverns at the sampled sites.

While 16S sequencing has provided essential insights into microbial diversity, the absence of functional analysis constrains our understanding of the metabolic roles within these communities. Future research endeavors should incorporate metagenomic and metatranscriptomic approaches to conduct a more comprehensive investigation of microbial functions and interactions. Furthermore, expanding seasonal sampling initiatives could elucidate temporal influences on microbial composition and ecosystem functionality.

In conclusion, this study demonstrates that the hot springs in northeastern Mexico host highly diverse microbial communities and represents the first comprehensive analysis of microbial diversity present in low-to medium-enthalpy hot springs, a geothermal region that remains largely unexplored in microbiological research. The presence of geochemical gradients, such as fluctuations in electrical conductivity, bicarbonates, sulfates, and hydrogen sulfide emissions, creates unique environmental niches, that foster specialized communities with essential metabolic functions vital for maintaining the ecological balance of these ecosystems. Moreover, possess significant potential for biotechnological applications. For example, genera associated with sulfur and hydrogen oxidation metabolic pathways, such as Sulfurovum and Hydrogenophaga, have demonstrated significant potential in bioremediation and detoxification processes involving sulfur compounds and heavy metals (Qattan, 2025). Additionally, the dominance of phyla such as Pseudomonadota and Chlorobiota indicates their adaptation to these extreme environments and underscores their ecological significance. These findings not only advance our understanding of microbial ecology in low-to medium-enthalpy geothermal systems, but they also emphasize the importance of conserving the microbiomes associated with these unique habitats. The observed high microbial diversity is intricately linked to the functional integrity of the springs, and any loss could jeopardize essential ecological processes, such as nutrient cycling and detoxification, while also potentially eradicating valuable genetic and metabolic resources. Therefore, safeguarding these microbial communities is crucial, not only for maintaining ecosystem stability, but also for preserving their biotechnological potential.

## Data Availability

The data presented in this study are deposited in the NCBI GenBank repository under BioProject accession number PRJNA1288561 (Microbiome at the springs of the Sierra Madre Oriental, Mexico). The BioProject includes the associated BioSamples with the following accession number ranges: SAMN49854367 to SAMN49854621, SAMN49854706 to SAMN49854883, SAMN49856174 to SAMN49856399, SAMN49856858 to SAMN49857210, SAMN49857745 to SAMN49858075, and SAMN49855763 to SAMN49855820. This Targeted Locus Study project has been deposited at DDBJ/EMBL/GenBank under the accession KJIH00000000. The version described in this paper is the first version, KJIH01000000.

## References

[ref1] Abed R. Koster J. (2005). The direct of aerobic heterotrophic bacteria associated with cyanobacteria in the degradation of oil compounds. Int. Biodeterior. Biodegradation 55, 29–37. doi: 10.1016/j.ibiod.2004.07.001

[ref2] Aliyu G. Ezugworie F. Onwosi C. Nnamchi C. Ekwealor C. Igbokwe V.. (2024). Multi-stress adaptive lifestyle of acidophiles enhances their robustness for biotechnological and environmental applications. Sci. Total Environ. 954:176190. doi: 10.1016/j.scitotenv.2024.176190, PMID: 39265677

[ref3] Badhai J. Ghosh T. Das S. (2015). Taxonomic and functional characteristics of microbial communities and their correlation with physicochemical properties of four geothermal springs in Odisha, India. Front. Microbiol. 6:1166. doi: 10.3389/fmicb.2015.01166, PMID: 26579081 PMC4620158

[ref4] Barosa B. Ferrillo A. Selci M. Giardina M. Bastianoni A. Correggia M.. (2023). Mapping the microbial diversity associated with different geochemical regimes in the shallow-water hydrothermal vents of the Aeolian archipelago, Italy. Front. Microbiol. 14:1134114. doi: 10.3389/fmicb.2023.1134114, PMID: 37637107 PMC10452888

[ref5] Beretta G. Sangalli M. Sezenna E. Tofalos A. E. Franzetti A. Saponaro S. (2024). Reducción electroquímica microbiana de Cr (VI) en un sistema de flujo continuo en el suelo. Eval. Gest. Amb. Integr. 20, 2033–2049. 10.1002/ieam.4972 38953765

[ref6] Bolyen E. Rideout J. R. Dillon M. R. Bokulich N. A. Abnet C. C. Al-Ghalith G. A.. (2019). Reproducible, interactive, scalable and extensible microbiome data science using QIIME 2. Nat. Biotechnol. 37, 852–857. doi: 10.1038/s41587-019-0209-9, PMID: 31341288 PMC7015180

[ref7] Briggs B. Brodie E. Tom L. Dong H. Jiang H. Huang Q.. (2014). Seasonal patterns in microbial communities inhabiting the hot springs of Tengchong, Yunnan Province China. Environ. Microbiol. 16, 1579–1591. doi: 10.1111/1462-2920.12311, PMID: 24148100

[ref8] Castelán-Sánchez H. Meza-Rodríguez P. Carrillo E. Ríos-Vázquez D. Liñan-Torres A. Batista-García R.. (2020). The microbial composition in circumneutral thermal springs from Chignahuapan, Puebla, Mexico reveals the presence of particular sulfur-oxidizing bacterial and viral communities. Microorganisms 8:1677. doi: 10.3390/microorganisms8111677, PMID: 33137872 PMC7692377

[ref9] Chan C. Chan K. Ee R. Hong K. Urbieta M. Donati E.. (2017). Effects of physiochemical factors on prokaryotic biodiversity in Malaysian circumneutral hot springs. Front. Microbiol. 8:1252. doi: 10.3389/fmicb.2017.01252, PMID: 28729863 PMC5498555

[ref10] Chao A. Hsieh T. (2016) iNEXT (iNterpolation and EXTrapolation) Online: Software for Interpolation and Extrapolation of Species Diversity. Available online at: http://chao.stat.nthu.edu.tw/wordpress/software_download/.

[ref11] Chao A. Jost L. (2012). Coverage-based rarefaction and extrapolation: standardizing samples by completeness rather than size. Ecology 93, 2533–2547. doi: 10.1890/11-1952.1, PMID: 23431585

[ref12] Cho S. Kimm MiHee K. Lee YoungOk L. (2016). Effect of pH on soil bacterial diversity. J. Ecol. Environ. 40:10. doi: 10.1186/s41610-016-0004-1

[ref13] Dong H. Huang L. Zhao L. Zeng Q. Liu X. Sheng Y.. (2022). A critical review of mineral–microbe interaction and co-evolution: mechanisms and applications. Natl. Sci. Rev. 9:nwac128. doi: 10.1093/nsr/nwac128, PMID: 36196117 PMC9522408

[ref14] Dufrêne M. Legendre P. (1997). Species assemblages and indicator species: the need for a flexible asymmetrical approach. Ecol. Monogr. 67, 345–366. doi: 10.1890/0012-9615(1997)067[0345,SAAIST]2.0.CO;2

[ref15] Edgar R. (2010). Search and clustering orders of magnitude faster than BLAST. Bioinformatics 26, 2460–2461. doi: 10.1093/bioinformatics/btq461, PMID: 20709691

[ref16] Edgar R. (2016). SINTAX: a simple non-Bayesian taxon-omy classifier for 16S and ITS sequences. Bio Rxiv. doi: 10.1101/074161

[ref17] Edgar R. Haas B. Clemente J. Quince C. Knight R. (2011). Uchime improves sensitivity and speed of chimera detection. Bioinformatics 27, 2194–2200. doi: 10.1093/bioinformatics/btr381, PMID: 21700674 PMC3150044

[ref18] Espinasa-Pereña R. Nieto-Torres A. (2015). Análisis de la vulnerabilidad a fenómenos kársticos: México, Technical Report. Secretaría de Gobernación, Coordinación Nacional de Protección Civil, Centro Nacional de Prevención de Desastres.

[ref19] Ghilamicael A. Budambula N. Anami S. Mehari T. Boga H. (2017). Evaluation of prokaryotic diversity of five hot springs in Eritrea. BMC Microbiol. 17:203. doi: 10.1186/s12866-017-1113-4, PMID: 28938870 PMC5610464

[ref20] Guo L. Wang G. Sheng Y. Shi Z. (2021). Hydrogeochemical constraints shape hot spring microbial community compositions: evidence from acidic, moderate-temperature springs and alkaline, high-temperature springs, southwestern Yunnan geothermal areas, China. J. Geophys. Res. Biogeosci. 126:e2020JG005868. doi: 10.1029/2020JG005868

[ref21] Hamady M. Lozupone C. Knight R. (2010). Fast Uni Frac: facilitating high-throughput phylogenetic analyses of microbial communities including analysis of pyrosequencing and PhyloChip data. ISME J. 4, 17–27. doi: 10.1038/ismej.2009.97, PMID: 19710709 PMC2797552

[ref22] Hou W. Wang S. Dong H. Jiang H. Briggs B. Peacock J.. (2013). A comprehensive census of microbial diversity in hot springs of Tengchong, Yunnan Province China using 16S rRNA gene pyrosequencing. PLoS One 8:e53350. doi: 10.1371/journal.pone.0053350, PMID: 23326417 PMC3541193

[ref23] Howells A. E. Leong J. A. Ely T. Santana M. Robinson K. Esquivel-Elizondo S.. (2022). Energetically informed niche models of hydrogenotrophs detected in sediments of serpentinized fluids of the Samail ophiolite of Oman. J. Geophys. Res. Biogeosci. 127:e2021JG006317. doi: 10.1029/2021JG006317

[ref24] Jost L. (2006). Entropy and diversity. Oikos 113, 363–375. doi: 10.1111/j.2006.0030-1299.14714.x

[ref25] Kruglikov A. Xia X. (2024). Mesophiles vs. thermophiles: untangling the hot mess of intrinsically disordered proteins and growth temperature of bacteria. Int. J. Mol. Sci. 25:2000. doi: 10.3390/ijms25042000, PMID: 38396678 PMC10889376

[ref26] López-Sandoval O. Montejano G. Carmona J. Cantoral E. Becerra-Absalón I. (2016). Diversidad algal de un ambiente extremo: el manantial geotermal Los Hervideros, México. Rev. Mex. Biodivers. 87, 1–9. doi: 10.1016/j.rmb.2016.01.004

[ref27] Madigan M. Martinko J. Parker J. (2021). Brock biology of microorganisms. 16th Edn. London, United Kingdom: Pearson.

[ref28] Madigan M. Schaaf N. Sattley W. (2017). “The Chlorobiaceae, Chloroflexaceae, and Heliobacteriaceae” in Modern topics in the phototrophic prokaryotes. ed. Hallenbeck P. (Cham: Springer).

[ref29] Martínez G. T. (2024). Aislamiento y caracterización de bacterias resistentes a altas temperaturas de las aguas termales San Francisco-Guayllabamba ubicadas en el cantón Chambo, provincia de Chimborazo. Dissertation/bachelor’s thesis. Riobamba: Escuela Superior Politécnica de Chimborazo.

[ref30] Martínez-Espinosa R. (2020). Microorganisms and their metabolic capabilities in the context of the biogeochemical nitrogen cycle at extreme environments. Int. J. Mol. Sci. 21:4228. doi: 10.3390/ijms21124228, PMID: 32545812 PMC7349289

[ref31] Medina-Chávez N. O. Rodríguez-Cruz U. E. Souza V. De la Torre-Zavala S. Travisano M. (2025). Salty secrets of *Halobacterium salinarum* AD88: a new archaeal ecotype isolated from Cuatro Cienegas Basin. BMC Genomics 26:399. doi: 10.1186/s12864-025-11550-9, PMID: 40275130 PMC12023398

[ref32] Merino N. Aronson H. Bojanova D. Feyhl-Buska J. Wong M. Zhang S.. (2019). Vivir en los extremos: extremófilos y los límites de la vida en un contexto planetario. Front. Microbiol. 10:1785. doi: 10.3389/fmicb.2019.01785 31456760 PMC6700686

[ref33] Moreno C. Barragán F. Pineda E. Pavón N. (2011). Reanálisis de la diversidad alfa: alternativas para interpretar y comparar información sobre comunidades ecológicas. Rev. Mex. Biodivers. 82, 1249–1261. doi: 10.22201/ib.20078706e.2011.4.745

[ref34] Narsing Rao M. P. Dong Z. Y. Luo Z. H. Li M. M. Liu B. B. Guo S. X.. (2021). Physicochemical and microbial diversity analyses of Indian Hot Springs. Front. Microbiol. 12:627200. doi: 10.3389/fmicb.2021.627200, PMID: 33763045 PMC7982846

[ref35] Ortega-Villar R. Escalante A. Astudillo-Melgar F. Lizárraga-Mendiola L. Vázquez-Rodríguez G. Hidalgo-Lara M.. (2024). Isolation and characterization of thermophilic bacteria from a hot spring in the state of Hidalgo, Mexico, and geochemical analysis of the thermal water. Microorganisms 12:1066. doi: 10.3390/microorganisms12061066, PMID: 38930448 PMC11205571

[ref36] Pantoja-Irys J. de la Rosa-Manzano E. Martínez-Ávalos J. Guerra-Pérez A. Mora-Olivo A. Arellano-Méndez L.. (2025). Diversity of plant communities surrounding the hot springs on the eastern flank of the Sierra Madre Oriental, northeastern Mexico. Biology 14:382. doi: 10.3390/biology14040382 40282247 PMC12025227

[ref37] Pantoja-Irys J. Mujica-Sánchez H. Arista-Cázares L. Hernández-García C. Wagner M. (2022). Environmental geology and isotopic evaluation of springs within the central part of the sierra Cerro de La Silla, northeastern México. J. S. Am. Earth Sci. 119:104017. doi: 10.1016/j.jsames.2022.104017

[ref38] Prieto-Barajas C. Alfaro-Cuevas R. Valencia-Cantero E. Santoyo G. (2017). Effect of seasonality and physicochemical parameters on bacterial communities in two hot spring microbial mats from Araró, Mexico. Rev. Mex. Biodivers. 88, 616–624. doi: 10.1016/j.rmb.2017.07.010

[ref39] Purcell D. Sompong U. Yim L. Barraclough T. Peerapornpisal Y. Pointing S. (2007). The effects of temperature, pH and sulphide on the community structure of hyperthermophilic streamers in hot springs of northern Thailand. FEMS Microbiol. Ecol. 60, 456–466. doi: 10.1111/J.1574-6941.2007.00302.X, PMID: 17386034

[ref40] Qattan S. Y. (2025). Harnessing bacterial consortia for effective bioremediation: targeted removal of heavy metals, hydrocarbons, and persistent pollutants. Environ. Sci. Eur. 37:85. doi: 10.1186/s12302-025-01103-y

[ref41] Rodríguez-Cruz U. E. Castelán-Sánchez H. G. Madrigal-Trejo D. Eguiarte L. E. Souza V. (2024). Uncovering novel bacterial and archaeal diversity: genomic insights from metagenome-assembled genomes in Cuatro Cienegas, Coahuila. Front. Microbiol. 15:1369263. doi: 10.3389/fmicb.2024.1369263, PMID: 38873164 PMC11169877

[ref42] Seo H. Kim J. Lee S. Lee S. (2024). The plant-associated Flavobacterium: a hidden helper for improving plant health. Plant Pathol. J. 40, 251–260. doi: 10.5423/PPJ.RW.01.2024.0019, PMID: 38835296 PMC11162857

[ref9001] Servicio Meteorológico Nacional (Mexico). (2021). National Meteorological Service [Internet]. Mexico City: National Water Commission (CONAGUA); [cited 2021]. Available from: https://smn.conagua.gob.mx/es/

[ref43] Sharp C. E. Brady A. L. Sharp G. H. Grasby S. E. Stott M. B. Dunfield P. F. (2014). Humboldt’s spa: microbial diversity is controlled by temperature in geothermal environments. ISME J. 8, 1166–1174. doi: 10.1038/ismej.2013.237, PMID: 24430481 PMC4030231

[ref44] Sorokin D. Berben T. Melton E. Overmars L. Vavourakis C. Muyzer G. (2014). Microbial diversity and biogeochemical cycling in soda lakes. Extremophiles 18, 791–809. doi: 10.1007/s00792-014-0670-9, PMID: 25156418 PMC4158274

[ref45] Sriaporn C. Campbell K. Van Kranendonk M. Handley K. (2023). Bacterial and archaeal community distributions and cosmopolitanism across physicochemically diverse hot springs. ISME Commun. 3:80. doi: 10.1038/s43705-023-00291-z 37596308 PMC10439147

[ref46] Sun Q. L. Xu K. Cao L. Du Z. Wang M. Sun L. (2023). Nitrogen and sulfur cycling driven by Campylobacterota in the sediment–water interface of deep-sea cold seep: a case in the South China Sea. MBio 14:e00117-23. doi: 10.1128/mbio.00117-23 37409803 PMC10470523

[ref47] Sun Q. L. Zhang J. Wang M. X. Cao L. Du Z. F. Sun Y. Y.. (2020). High-throughput sequencing reveals a potentially novel Sulfurovum species dominating the microbial communities of the seawater–sediment interface of a deep-sea cold seep in South China Sea. Microorganisms 8:687. doi: 10.3390/microorganisms8050687, PMID: 32397229 PMC7284658

[ref9002] Susanti R. Listiaji P. Mukaromah R. L. Nisa F. F. Kenarni N. R. Jaya A. F. (2025). Microbial Diversity in Hot Spring Soil Microbiome. Biosaintifika, 17, 80–90.

[ref48] Tang H. Xiang G. Xiao W. Yang Z. Zhao B. (2024). Microbial mediated remediation of heavy metals toxicity: mechanisms and future prospects. Front. Plant Sci. 15:1420408. doi: 10.3389/fpls.2024.1420408, PMID: 39100088 PMC11294182

[ref49] Thai T. D. Lim W. Na D. (2023). Synthetic bacteria for the detection and bioremediation of heavy metals. Front. Bioeng. Biotechnol. 11:1178680. doi: 10.3389/fbioe.2023.1178680, PMID: 37122866 PMC10133563

[ref50] Valcheva N. Ignatov I. Dinkov G. (2020). Microbiological and physicochemical research of thermal spring and mountain spring waters in the district of Sliven, Bulgaria. J. Adv. Microbiol. 20, 9–17. doi: 10.9734/jamb/2020/v20i230213

[ref51] Von Hegner I. (2020). Extremophiles: a special or general case in the search for extra-terrestrial life? Extremophiles 24, 167–175. doi: 10.1007/s00792-019-01144-1, PMID: 31707497

[ref52] Wang X. Pecoraro L. (2021). Diversity and co-occurrence patterns of fungal and bacterial communities from alkaline sediments and water of Julong high-altitude Hot Springs at Tianchi volcano, Northeast China. Biology 10:894. doi: 10.3390/biology10090894, PMID: 34571771 PMC8464750

[ref53] Wang J. Zheng Q. Wang S. Zeng J. Yuan Q. Zhong Y.. (2023). Characterization of two novel chemolithoautotrophic bacteria of *Sulfurovum* from marine coastal environments and further comparative genomic analyses revealed species differentiation among deep-sea hydrothermal vent and non-vent origins. Front. Mar. Sci. 10:1222526. doi: 10.3389/fmars.2023.1222526

[ref54] Weiland-Bräuer N. (2021). Friends or foes—microbial interactions in nature. Biology 10:496. doi: 10.3390/biology10060496, PMID: 34199553 PMC8229319

[ref55] Whaley-Martin K. J. Chen L. X. Nelson T. C. Gordon J. Kantor R. Twible L. E.. (2023). O2 partitioning of sulfur oxidizing bacteria drives acidity and thiosulfate distributions in mining waters. Nat. Commun. 14:2006. doi: 10.1038/s41467-023-37426-8, PMID: 37037821 PMC10086054

